# Corticosterone: a costly mediator of signal honesty in sand lizards

**DOI:** 10.1002/ece3.2318

**Published:** 2016-09-28

**Authors:** Willow R. Lindsay, Erik Wapstra, Bengt Silverin, Mats Olsson

**Affiliations:** ^1^ Department of Biological and Environmental Sciences Göteborg University Göteborg Sweden; ^2^ School of Biological Sciences University of Tasmania Hobart Tas. Australia; ^3^ School of Biological Sciences University of Sydney Sydney NSW Australia

**Keywords:** Badge size, coloration, ectoparasite, endoparasite, glucocorticoids, *Lacerta agilis*, lizard

## Abstract

The mechanisms underlying honest signal expression remain elusive and may involve the integration of social and physiological costs. Corticosterone is a socially modulated metabolic hormone that mediates energy investment and behavior and may therefore function to deter dishonest signal expression. We examined the relationship between corticosterone and green badge coloration in male sand lizards (*Lacerta agilis*), hypothesizing that physiological and behavioral costs resulting from elevated baseline glucocorticoids function in maintenance of honest signal expression. We found that large‐badged males had higher corticosterone titer, with this relationship apparent at the end of the season and absent early in the season. Large‐badged males also suffered higher ectoparasite load (number of tick nymphs), despite being in better condition than small‐badged males. Ectoparasite load was positively related to corticosterone titer early in the season at the time of badge formation. High‐condition individuals had lower corticosterone and lower numbers of ectoparasites than low‐condition individuals, suggestive of conditional variation in ability to withstand costs of corticosterone. We found an opposing negative relationship between corticosterone titer and endoparasite load. Corticosterone titer was also negatively associated with male mobility, a fitness‐determining behavior in this species. Because badge size is involved in mediating agonistic social interactions in this species, our results suggest that badge‐dependent variation in corticosterone is likely reflective of variation in social conditions experienced over the course of the season. Our results implicate corticosterone in maintenance of signal honesty, both early in the season through enforcement of physiological costs (ectoparasite load) and during the season through behavioral costs (male mobility). We propose that socially modulated variation in corticosterone critically functions in mediation of signal honesty without requiring a direct role for corticosterone in trait expression.

## Introduction

Sexually selected ornaments accurately reflect individual “quality” in most systems (Searcy and Nowicki 2005), yet the mechanisms linking condition to signal expression remain elusive. Two classes of costly mechanisms have been investigated: hormones that mediate trait expression through trade‐offs in investment of expensive resources toward self‐maintenance versus signal production, and social costs of trait elaboration accrued through aggressive challenges from receivers (Rohwer 1977). Due to the dynamic, bidirectional feedback between social behavior and hormone production (Hirschenhauser and Oliveira 2006; Rubenstein and Hauber 2008; Creel et al. 2013), social and physiological regulation of signal honesty should not be studied in exclusion (Tibbetts 2014; Vitousek et al. 2014).

Ornamental color patches often function to signal social status, relaying information about an individual's competitive ability and coordinating the frequency and outcome of socially antagonistic interactions (Senar 2006). In turn, social aggression is closely tied to fluctuations in glucocorticoid stress hormones (reviewed by Creel 2001; Creel et al. 2013), and thus, ornamental color patches may predict glucocorticoid titer. To illustrate, artificial enhancement of the white plumage patch on the crowns of *Zonotrichia leucophrys gambelii* (white‐crowned sparrows) induced both an increase in aggression from challengers and an increase in baseline corticosterone (CORT, the primary glucocorticoid in avian, amphibian, and reptilian species; Laubach et al. 2013). In this example, CORT may mediate behavioral and physiological costs of dishonest signaling (artificial signal enhancement). Several other studies, however, document positive covariation between CORT and sexual and status signaling traits including avian beak and plumage color (McGraw et al. 2011; Lendvai et al. 2013) and lizard belly color (Fitze et al. 2009; Cote et al. 2010). Molecular mechanisms linking CORT with coloration remain unclear, and this link may instead involve complex interactions with a correlated third variable, social dominance (Fitze et al. 2009; Cote et al. 2010). Dominant individuals often express higher baseline glucocorticoid titer than do subordinate individuals, likely as a consequence of engagement in more frequent aggressive encounters (Creel 2001; Creel et al. 2013). As such, dominant males with large ornaments may experience higher relative glucocorticoid titer than less ornamented, subordinate individuals. This hypothesis remains to be tested and is interesting in light of physiological trade‐offs orchestrated via glucocorticoids which may function to deter dishonest signaling (Husak and Moore 2008).

Glucocorticoid hormones mobilize energy stores and adaptively adjust behavior in response to changing social and environmental conditions (Sapolsky et al. 2000). These hormones are crucial for maintaining homeostasis during the physiological stress response, in part by downregulating expensive self‐maintenance systems (Romero 2002, 2004; Wingfield and Sapolsky 2003). Specifically, chronically elevated glucocorticoids can inhibit the hypothalamic–pituitary–gonadal axis (Sapolsky et al. 2000). This can have negative effects on testosterone titer (T), an important suppressive effect when signaling traits rely on T for either production or maintenance. Glucocorticoid‐induced immune suppression can also increase parasite load (Barnard et al. 1996, 1998; Belden and Kiesecker 2005; Chandramathi et al. 2014), an important and potentially honesty‐enforcing cost in light of the much‐investigated link between parasite resistance and degree of signaling trait elaboration (Hamilton and Zuk 1982; Folstad and Karter 1992). Finally, elevated CORT may redirect energy usage away from current reproductive efforts toward behaviors necessary to ensure survival (Wingfield et al. 1998; Wingfield and Romero 2001; Moore and Jessop 2003) with possible impacts on reproductive success (Bonier et al. 2009). Despite potential costs to immune function, parasite resistance, and reproduction, moderate and short‐term elevations of glucocorticoids can facilitate energy expenditure toward production of metabolically demanding reproductive behaviors, maintenance of social rank, and ornamental characters (reviewed by Sapolsky et al. 2000; Moore and Jessop 2003; Leary and Knapp 2014). Indeed, baseline glucocorticoids tend to be elevated during reproduction in many bird species, amphibians, and reptiles (Moore et al. 2000; Romero 2002; Moore and Jessop 2003). Thus, the advantages to glucocorticoid elevation when expressed by high‐status (and ostensibly high‐condition) individuals may balance potential costs (Barnard et al. 1996), making it difficult to predict directionality in the relationship between CORT and ornamentation.

Evidence to date linking glucocorticoids to ornamental trait expression is mixed [negative associations (Leary et al. 2006; Roulin et al. 2008; Wada et al. 2008; Mougeot et al. 2010); positive associations (Cote et al. 2010; McGraw et al. 2011; Lendvai et al. 2013)]. Hypotheses that predict a role for glucocorticoids in mediation of signal honesty often rely on the intermediary function of glucocorticoids in linking body condition to T titer (Husak and Moore 2008; Rubenstein and Hauber 2008). However, glucocorticoids can have direct and T‐independent effects on trait expression (Leary and Knapp 2014 and references therein), and many signaling traits themselves are unresponsive to T (Owens and Short 1995; Roberts et al. 2004; Olsson et al. 2013). The role of glucocorticoids in mediation of signal expression may instead involve a complex interaction between degree of ornamentation, social behavior, and physiological costs arising from elevated levels of baseline glucocorticoids.

**Figure 1:**
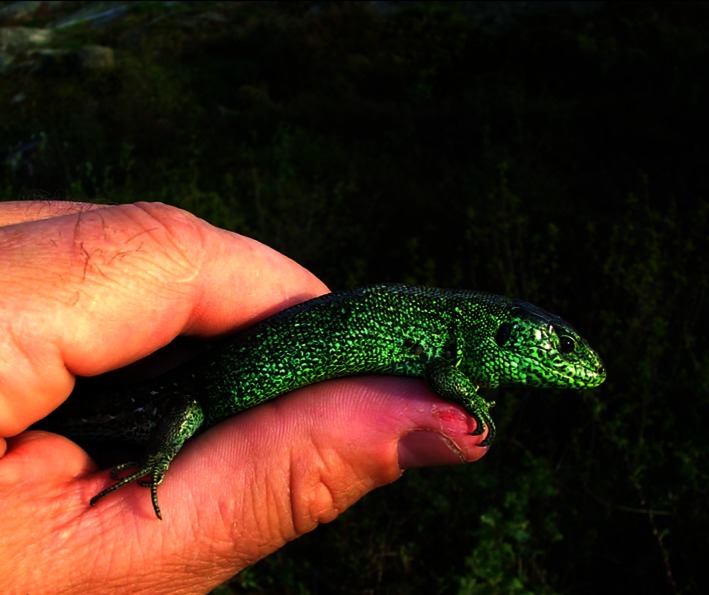
Male sand lizard (*Lacerta agilis*) with a green ventral badge and three ectoparasite ticks adjacent to the foreleg. Photograph by Erik Wapstra.

Here, we examined the relationship between CORT and condition‐dependent green badge coloration in male sand lizards (*Lacerta agilis*). The bright green “badge,” present on the sides of male sand lizards during the breeding season (Fig. 1), is an agonistic signal of male fighting ability and may therefore be related to hormone titer. Males with experimentally enhanced badges are more likely to win a staged contest (Olsson 1994a; Olsson et al. 2005), and natural variation in badge size is related to the probability of engaging in aggressive interactions [as indicated by the number of head scars accrued over the course of the breeding season (Olsson et al. 2005)]. Despite potential costs of aggression, large‐badged males have higher reproductive success (Olsson 1994a; Olsson et al. 2000, 2005; Anderholm et al. 2004) and are in better body condition (Olsson 1994a; Anderholm et al. 2004). The correlation between condition and badge size does not appear to be mediated by T as neither circulating concentrations of T nor artificially elevated T are related to badge size (Olsson and Silverin 1997; Olsson et al. 2000). Instead, the social costs of fighting, felt disproportionally by small males, may deter cheating (Olsson 1994b). Specifically, small males with larger‐than‐average badges experience greater declines in body mass and show a trend for lower survival than larger males of equal badge size (Olsson 1994b; Anderholm et al. 2004).

We hypothesize that physiological and behavioral costs related to elevated baseline glucocorticoids (i.e., CORT titer) function in maintenance of honest signal expression in male sand lizards. We examine covariation between badge size, CORT, and costly parasite load both early in the season, at the time of badge development, and late in the season, when we assume social dominance hierarchies to be well established. By doing so, we aim to differentiate between potential direct effects of CORT on trait production vs. indirect associations with trait intensity arising from a correlated third variable, social aggression. We ask (1) whether badge size is related to baseline CORT and predictive of costly parasite infection; (2) whether variation in body condition and both ecto‐ or endoparasite load are related to CORT, indicative of physiological costs of elevated CORT, and whether these potential costs are experienced equally by high‐ versus low‐condition individuals; and (3) whether elevated CORT is behaviorally costly, related to expression of fitness‐enhancing reproductive behavior (male mobility). More mobile males are expected to encounter and acquire a greater number of mates. As a consequence, mobility positively predicts reproductive success in this species (Olsson et al. 2000), and would likely come at a cost of increased detectability by areal predators. We therefore predict a negative relationship between CORT and male mobility if elevated baseline CORT selectively shunts energy expenditure toward survival and away from behaviors involved in reproduction (Wingfield et al. 1998; Wingfield and Romero 2001; Moore and Jessop 2003).

## Materials and Methods

Here, we provide a brief description of methodology as details of the field methods have been published elsewhere (Madsen et al. 2000; Olsson et al. 2000). Males were captured by noose soon after emergence from hibernation at our study site at Asketunnan, 50 km south of Gothenburg on the Swedish west coast. The male color badge is produced 1–2 weeks after emergence from hibernation, and was therefore complete or nearing completion at the time of capture. Blood samples were collected from the *sinus angularis* within 30 sec of capture, and thus, our measurement of CORT does not reflect the stress of capture and handling. All animals were measured (snout–vent length, total length) and weighed to the nearest 0.001 g. Males were scanned on a flatbed scanner, and badge size was estimated as the proportion of green coloration versus the remaining side of the body (Olsson and Madsen 2001).

Morphological variables and ectoparasite counts used in this study stem from 10 years of data (1998–2007) collected on our field population, while measurements of endoparasite counts and male behavior (mobility) are restricted to samples collected in 1998. Plasma CORT concentrations were measured in 1998 and in 2004 and, as with morphological variables, were collected from individuals at the time of first observation in the population each season. We therefore classified these as “early‐season” measurements of CORT. To determine ectoparasite load, we counted the number of nymphs of the tick *Ixodes ricinus* (Olsson 1992) which gradually accumulate on the surface of the skin through the season beginning after emergence from hibernation. As a measure of endoparasite load, we counted the number of haemoprotid parasites in whole blood samples (following the methods of Olsson et al. 2005).

Males sampled in 1998, the only year for which we obtained two measurements of CORT, badge size, ectoparasite load, and body condition (early‐ and late‐season), were involved in a hormone manipulation experiment. On the day following capture, males were anaesthetized and given either an empty silastic control implant (*N *=* *21) or a silastic implant containing crystallized testosterone (*N *=* *21; 4 mm effective length: Sigma product no. T 1500; Olsson et al. 2000). All males were marked on their backs for individual identification with a numbered cloth adhesive sticker, blood was taken for hormone measurement (early‐season), and males were released at the place of capture. We visually monitored active males (from >2 m) daily for the duration of the approximately 4‐ to 5‐week breeding period, at the end of which they were recaptured and measured, and a second blood sample collected (late‐season). A measure of male “mobility,” the accumulated distance in meters between points each male was sighted, was taken over the entirety of the breeding phase (Olsson et al. 2000). We have no indication that animals were disturbed by our presence.

### Corticosterone radioimmunoassay

Plasma CORT levels for samples collected in 1998 were measured with a single radioimmunoassay (RIA) at Gothenburg University following protocols established by B. Silverin (Silverin 1997; Silverin et al. 1997; Cockrem and Silverin 2002); for detailed methods for the sand lizard samples, see Olsson et al. (2005). A direct radioimmunoassay (without column chromatography) was applied according to Wingfield et al. (1992). In brief, plasma samples (10–20 *μ*L) were spiked with tritiated CORT for recovery determination and diluted with distilled water to a total volume of 400 *μ*L. Samples were then extracted overnight with 4 mL redistilled dichloromethane, dried under nitrogen, and resuspended in 500 *μ*L phosphate‐buffered saline. Samples were assayed in duplicate, and CORT recovery was 88–95% after extraction.

Samples collected in 2004 were measured at University of Wollongong in a single assay following a similar direct RIA protocol (Wingfield et al. 1992; see also Buttemer et al. 2015). As above, plasma samples (100 *μ*L) were spiked with tritiated CORT for recovery determination and extracted with dichloromethane. Dried and phosphate‐buffered saline reconstituted samples were assayed in duplicate, and CORT measurements were within the range of detectability. All samples were corrected for an average CORT recovery of 87.5%.

### Statistics

Analyses were conducted on two discrete datasets: early‐season measurements of morphology, ectoparasite load, and CORT (combined across sampling years: 1998 *N *=* *39, 2004 *N *=* *124), and late‐season measurements of morphology, ectoparasite and endoparasite load, male mobility, and CORT (restricted to collections made in 1998, *N *=* *29). We additionally assessed the relationship between body condition, ectoparasite load, and badge size across our 10‐year observational dataset (*N *=* *336). Early‐season measurements of ectoparasite load, body condition (calculated as the residuals of a regression of log‐transformed mass on log‐transformed snout–vent length), badge size, and CORT taken during 1998 were assessed at the beginning of experimentation and are thus unaffected by treatment (T vs. control). The potential influence of T treatment on all late‐season measurements has been statistically accounted for by inclusion (and subsequent exclusion if nonsignificant) in statistical models. Specifically, T treatment had no effect on late‐season badge size, body condition, CORT, or ectoparasite load (all *P *>* *0.14), and thus, these data have been combined across treatments (see also Olsson et al. 2000, 2005). Treatment has been included as a factor in models of late‐season endoparasite load and male mobility (Table 1).

**Table 1:** Analyses of relationships in two discrete datasets: early‐season measures of badge size, parasitism, and CORT, and late‐season measures of badge size, parasitism, male mobility, and CORT

	Predictors	*F*	df	*P*
Early‐season				
Badge size	CORT	0.30	1,106	0.586
	Ectoparasite load	16.17	1,106	**0.0001**
	Body condition	5.88	1,106	**0.017**
	Date of capture	7.23	1,106	**0.008**
Ectoparasite load	CORT	15.94	1,137	**<0.0001**
	Body condition	2.53	1,137	0.114
	CORT * condition	6.58	1,137	**0.011**
Late‐season				
Badge size	CORT	4.59	1,24	**0.043**
	Ectoparasite load	4.97	1,24	0.036
Ectoparasite load	CORT	2.85	1,25	0.104
	Body condition	4.47	1,25	0.045
Endoparasite load	CORT	14.12	1,22	**0.001**
	Treatment	3.7	1,22	0.067
	Treatment*CORT	3.20	1,22	0.087
Male mobility	CORT	6.63	1,24	**0.017**
	Treatment	7.75	1,24	**0.010**

Only factors and interaction terms included in the final best‐fit models are provided. *P*‐values listed in bold represent factors that remained significant following FDR correction for multiple comparisons.

Both early‐season CORT and ectoparasite load differed between years (CORT *F*_1,161_ = 30.36, *P *<* *0.0001; ectoparasite load *F*_9,398_ = 2.19, *P *=* *0.022) and have therefore either been standardized for year or the effect of year has been controlled for by inclusion of year as a random factor in a mixed‐effect model. Time of capture and blood sampling was recorded for early‐season samples collected in 1998 only and was not correlated with measurements of CORT (*r*_s_ = −0.174, *P *=* *0.303, *N *=* *37). Date of capture is included as a potential predictive factor in analyses of early‐season badge size, but has no detectable effect on late‐season measurements of badge size (*r*_s_ = −0.205, *P *=* *0.268, *N *=* *31) and has therefore been excluded. Date of capture also had no influence on early‐ and late‐season CORT nor on early‐season ectoparasites. In order to meet assumptions of normality, we log‐transformed both early‐ and late‐season CORT, ectoparasite load, and badge size (Shapiro–Wilk's statistics, all *P *>* *0.08).

We used mixed‐effect models or generalized linear models (GLMs) with stepwise backward elimination to select the best fitted minimal models, eliminating factors and interactions at *P *>* *0.15. We examined the association between badge size, body condition, and ectoparasite load across all 10 years of observation using a model including individual ID and year as random factors and date of sampling as a covariate. All significant predictive factors and interactions for remaining models are provided in Table 1.

We adjusted for multiple comparisons within each group of analyses (early‐ and late‐season response variables of badge size, ectoparasite load, endoparasite load, and male mobility) using the false discovery rate procedure (FDR; Benjamini and Hochberg 1995). We present significance as generated through each GLM analysis and note instances where detected relationships are no longer significant following FDR correction (Table 1). All statistics were performed in SAS 9.4. (SAS Institute, Cary, NC, USA)

## Results

### Costs of badge size: parasite load and corticosterone

**Figure 2:**
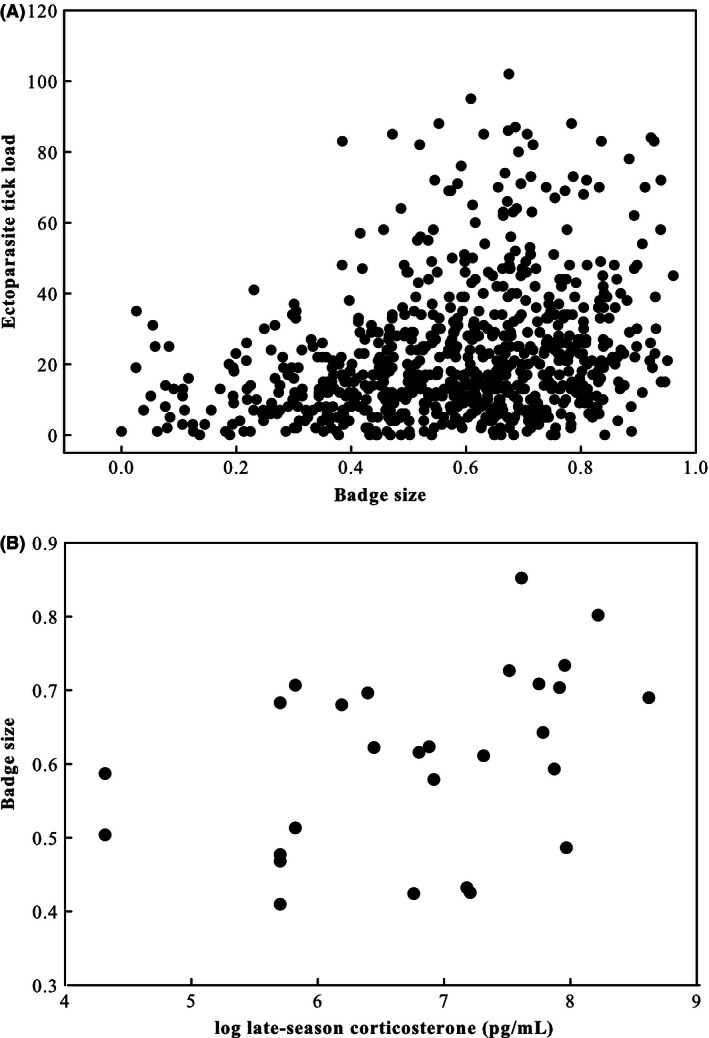
Positive relationships between badge size and numbers of ectoparasite ticks (A) and between late‐season badge size and log‐transformed CORT (B).

Badge size was positively associated with body condition (*F*_1,334_ = 12.42, *P *=* *0.0005) (see also Olsson 1994a; Olsson et al. 2000, 2005; Anderholm et al. 2004), but larger‐badged males suffered greater ectoparasite load (Fig. 2A; *F*_1,331_ = 25.57, *P *<* *0.0001). Larger‐badged males also had higher CORT late in the breeding season, but there was no relationship between CORT and badge size early in the season (Table 1; Fig. 2B). Concentrations of CORT measured early in the season did not predict late‐season CORT (*r*_s_ = 0.12, *P *=* *0.55, *N *=* *27).

### Costs of corticosterone: parasite load and male mobility

**Figure 3:**
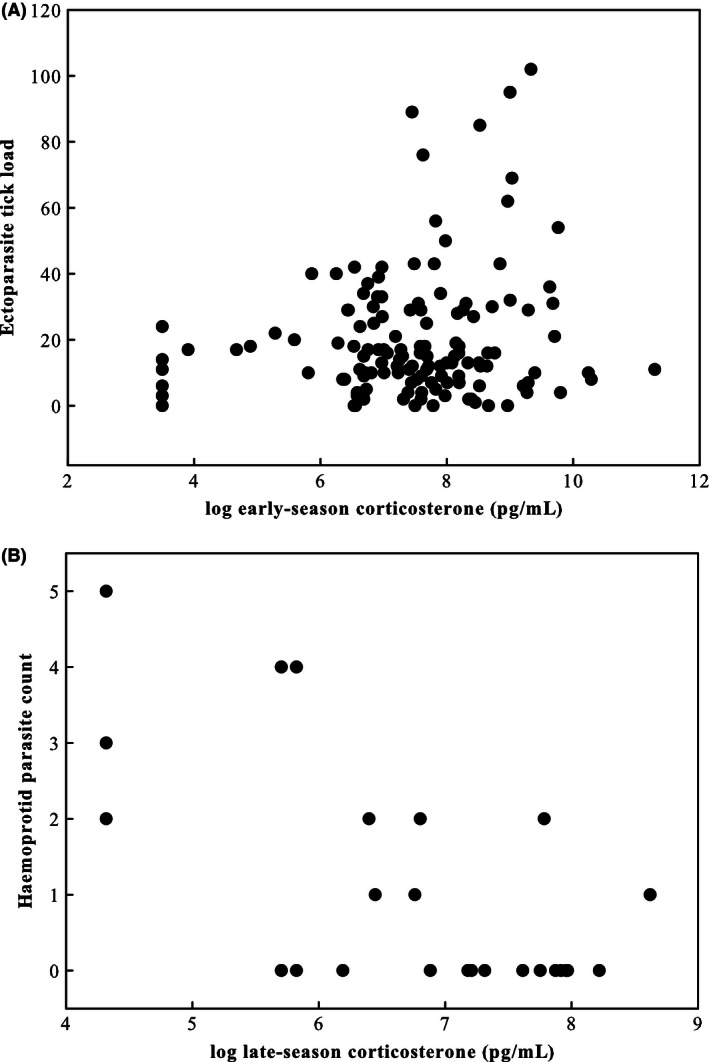
Positive relationship between log‐transformed early‐season CORT and ectoparasite tick number (A) and negative relationship between log‐transformed late‐season CORT and haemoprotid endoparasite load (B).

We found a positive relationship between early‐season CORT and ectoparasite load (Table 1; Fig. 3A). Interestingly, ectoparasite load was unrelated to body condition. However, we found a significant interaction between early‐season CORT and body condition on ectoparasite load such that high‐condition individuals tended to have lower CORT and lower numbers of ectoparasites than low‐condition individuals. Late‐season ectoparasite load was unrelated to late‐season CORT and body condition (Table 1).

Late‐season CORT was negatively related to endoparasite load (Table 1; Fig. 3B). There was a trend for T treated males to have a greater number of endoparasites than controls. There was also a trend for an interaction between late‐season CORT and treatment such that the negative association between CORT and endoparasite load was more apparent in individuals with high exogenous T.

Neither early‐ nor late‐season CORT varied with body condition (early‐season CORT * early‐season condition *r*_s_ **= 0.069, *P *=* *0.411, *N *=* *163; late‐season CORT * late‐season condition *r*_s_ **= −0.073, *P *=* *0.725, *N *=* *27).

**Figure 4:**
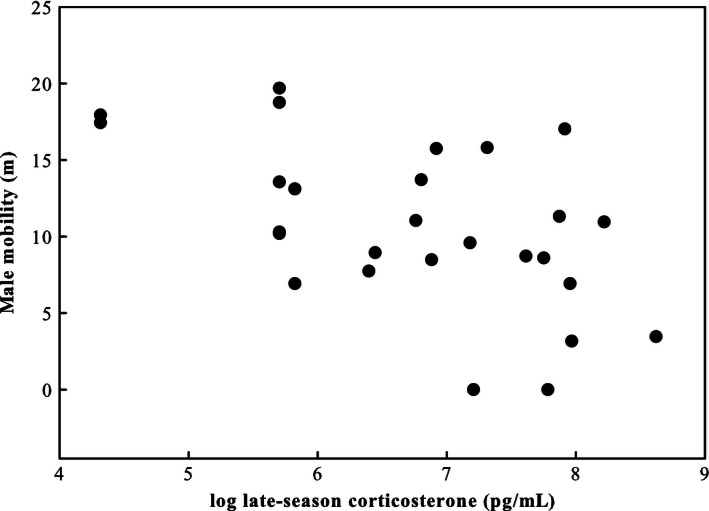
Negative relationship between log‐transformed late‐season CORT and male mobility.

Male mobility was negatively related to late‐season CORT (Table 1; Fig. 4) even after correcting for the strong positive effect of testosterone treatment on male mobility (see also Olsson et al. 2000). Male mobility was not related to either early‐season CORT (*F*_1,22_ = 1.55, *P *=* *0.226) nor to badge size (*r*_s_ = −0.102, *P *=* *0.604).

## Discussion

While carrying a large badge appears to be costly for male sand lizards in terms of associations with elevated CORT and ectoparasite load, only males in good condition produce large badges (Olsson 1994a; Anderholm et al. 2004). The physiological costs of elevated CORT, shown here as an increase in ectoparasite load, appear to be differentially experienced based on male condition such that high‐condition individuals accumulate fewer ectoparasites than low‐condition individuals at elevated levels of CORT. These data support predictions for handicap models of honest signal mediation (Zahavi 1975; Folstad and Karter 1992) (Zahavi 1975; Grafen 1990; Folstad and Karter 1992), where signal honesty is maintained via costs that vary based on the quality of the signaler (Grafen 1990). Elevated baseline CORT is also behaviorally costly, associated with reductions in the distance a male moved during the course of the breeding season. Male mobility positively predicts reproductive success in this (Olsson et al. 2000) and other species (Keogh et al. 2012; reviewed in Olsson and Madsen 1998). However, because mobility is not associated with badge size, the behavioral costs of elevated baseline CORT are likely felt equally by large‐ and small‐badged males. This is a cost that large‐badged males overcome, based on their higher reproductive success (Olsson 1994a; Olsson et al. 2000, 2005; Anderholm et al. 2004). Badge‐dependent variation in CORT is apparent only late in the season and thus likely arises from variation in social conditions experienced over the course of the season. Specifically, a dynamic feedback between male–male competitive interactions and glucocorticoid production may explain why larger‐badged males who engage in and win more agonistic interactions (Olsson 1994a; Olsson et al. 2005) have higher baseline CORT at the end of the season. Our results provide correlational evidence for CORT as a costly mediator of honest signal expression in male sand lizards, with variation likely arising as a consequence of badge size‐specific agonistic behaviors expressed over the course of the breeding season.

Larger‐badged males have more ectoparasites (see also Halliday et al. 2014; but see Molnár et al. 2012), and CORT appears to be an important mediator of this relationship. According to the Hamilton and Zuk hypothesis (Hamilton and Zuk 1982), ornamental traits should reflect underlying resistance to parasites as parasite load has significant impacts on the health and fitness of the host (Møller 1997). Building from this concept, the immunocompetence handicap hypothesis (Folstad and Karter 1992) proposed that the suppressive effects of T on the immune system (and thereby parasite abundance) and simultaneous associations with elaborate trait production should allow only high quality individuals to produce the best ornaments. This hypothesis can be extended to CORT (Møller 1995) without requiring a role for CORT in trait production. If, instead, CORT is associated with trait expression (through social modulation), costly CORT‐induced alterations in parasite load may act to ensure signal honesty, as may be the case here.

Interestingly however, our results indicate that the costs of CORT on susceptibility to parasite infection are not consistent between endo‐ and ectoparasites, corroborating other studies documenting differing relationships between parasite type and hormone titer (Barnard et al. 1996; Fuxjager et al. 2011). CORT titer is related to elevated ectoparasite load in sand lizards (this study) and other organisms (Barnard et al. 1996, 1998; Oppliger et al. 1998; Belden and Kiesecker 2005; Chandramathi et al. 2014; but see Hanley and Stamps 2002). We show an opposing negative relationship between CORT and sand lizard haemoprotid endoparasite load (see also Hanley and Stamps 2002). This is a surprising finding as ticks act as vectors for haemoprotids (Rheichenbach‐Klinke and Elkan 1965) and are therefore predicted to covary with traits that dictate tick load (e.g., hormone titer). This negative relationship may reflect the actions of exogenous dosages of T, where elevated T appears to offset costs of simultaneously elevated CORT. Opposing as well as additive effects of CORT and T on parasite abundance have been documented elsewhere (Barnard et al. 1996; Bortolotti et al. 2009), and along with our findings suggest caution in interpretation of studies based on measurements of a single hormone or parasite. Further research is necessary to determine causality in the relationship between CORT and parasite abundance and to investigate whether either ecto‐ or endoparasite infestation influences fitness in sand lizards.

Our results indicate that CORT may mediate physiological costs related to badge size without mediation of badge size itself, as the correlation between CORT and badge size only arises at the end of the season. A trade‐off between growth rate and degree of pigmentation in sand lizards (Olsson 1994b; Olsson and Silverin 1997) indicates that production of the badge may be energetically costly. The lack of association between CORT and badge size early in the season may therefore result from a downregulation of the glucocorticoid stress response during a period of expensive trait formation as appears to be the case for molt in birds (Romero 2002; but see Buttemer et al. 2015). However, this is unlikely, as we do not detect differences in CORT between early‐ and late‐season sampling periods.

CORT has direct and largely negative effects on color production (Calisi and Hews 2007; San‐Jose and Fitze 2013; Weiss et al. 2013) and, in particular, interferes with the process of melanogenesis through competitive binding of the melanocortin receptor necessary for melanin pigmentation (Ducrest et al. 2008; Roulin et al. 2008). In contrast, CORT treatment positively affects carotenoid‐dependent red coloration in three species: *Lacerta vivipara* (common lizard) (Cote et al. 2006; Fitze et al. 2009), *Taeniopygia guttata* (zebra finch) (McGraw et al. 2011), and *Haemorhous mexicanus* (house finch) (Lendvai et al. 2013). The mechanisms involved remain elusive, although CORT‐induced increases in metabolism and plasma carotenoid transport have been suggested (McGraw et al. 2011; Lendvai et al. 2013), but not experimentally supported (Fitze et al. 2009). Alternatively, the link between CORT and coloration may be driven by ornament‐dependent variation in social and agonistic behaviors. In this scenario, the ornament itself may be cheap to produce but have high social costs that are mediated by elevated hormone levels (Rubenstein and Hauber 2008; Tibbetts 2014).

Social aggression tends to increase glucocorticoids (reviewed by Creel 2001; Creel et al. 2013; but see DeNardo and Licht 1993; Barnard et al. 1994; Hanley and Stamps 2002), with differences between species in whether the dominant or the subordinate individual expresses higher CORT (Creel 2001; Creel et al. 2013). A strong relationship between badge size and frequency (or intensity) of aggression likely explains the elevated CORT in large‐badged sand lizard males. Large‐badged males win more staged contests (Olsson 1994a), but also have more fighting scars (Olsson et al. 2005), indicating either participation in more fights than small‐badged males or greater escalation of these aggressive encounters. While it is possible that the small‐badged males have a more elevated glucocorticoid stress response during an aggressive interaction, the more frequent engagement in aggression experienced by large‐badged individuals may lead to overall higher baseline glucocorticoids, as has been shown in other species (Creel et al. 2013). The lack of association between CORT and our index of body condition supports our hypothesis that the CORT–badge size relationship arises from variation in social rather than physiological condition; however, manipulation experiments are needed to verify this relationship.

While CORT may promote (and result from) social aggression, it appears to direct energy usage away from a behavior that functions primarily in mate acquisition (male mobility). The negative correlation between CORT and male mobility presented here is similar to findings in some (DeNardo and Sinervo 1994; Ricciardella et al. 2010), but not all reptilian and amphibian systems (Hanley and Stamps 2002; Cote et al. 2006), and is counter to the prediction that CORT serves to stimulate general locomotor activity (Breuner et al. 1998; Romero 2002). Our results instead hint at the presence of a CORT‐induced trade‐off between involvement in social aggression and investment in mobility, a behavior related to enhanced reproductive output (Olsson et al. 2005).

We add to a growing body of literature implicating a role for glucocorticoids in mediation of signal honesty (Rubenstein and Hauber 2008; Bortolotti et al. 2009; Weiss et al. 2013; Leary and Knapp 2014; Tibbetts 2014; Vitousek et al. 2014). Our findings show a positive relationship between CORT and badge size that likely reflects ornament‐dependent variation in social behaviors expressed over the course of the breeding season rather than direct effects of CORT on trait production. As such, signal honesty may be mediated via physiological and behavioral costs (increased ectoparasite load and decreased fitness‐enhancing mobility) generated through a dynamic feedback between social behavior and CORT production. The nature of the relationship between social aggression and baseline CORT in sand lizards requires further investigation.

## Data Accessibility

Data will be deposited in Dryad Digital Repository doi: 10.5061/dryad.67136

## Conflict of Interest

None declared.
